# Software-Based Three-Dimensional Deconvolution Microscopy of Cytoskeletal Proteins in Cultured Fibroblast Using Open-Source Software and Open Hardware †

**DOI:** 10.3390/jimaging5120088

**Published:** 2019-11-23

**Authors:** Kazuo Katoh

**Affiliations:** Faculty of Health Sciences, Tsukuba University of Technology, Laboratory of Human Anatomy and Cell Biology, 4-12-7 Kasuga, Tsukuba-city, Ibaraki 305-8521, Japan; katoichi@k.tsukuba-tech.ac.jp

**Keywords:** deconvolution microscopy, cell biology, actin filament, focal adhesion, image analysis

## Abstract

As conventional fluorescence microscopy and confocal laser scanning microscopy generally produce images with blurring at the upper and lower planes along the *z*-axis due to non-focal plane image information, the observation of biological images requires “deconvolution.” Therefore, a microscope system’s individual blur function (point spread function) is determined theoretically or by actual measurement of microbeads and processed mathematically to reduce noise and eliminate blurring as much as possible. Here the author describes the use of open-source software and open hardware design to build a deconvolution microscope at low cost, using readily available software and hardware. The advantage of this method is its cost-effectiveness and ability to construct a microscope system using commercially available optical components and open-source software. Although this system does not utilize expensive equipment, such as confocal and total internal reflection fluorescence microscopes, decent images can be obtained even without previous experience in electronics and optics.

## 1. Introduction

Epifluorescence microscopy can effectively image cytoskeletal proteins and organelles in cultured cells. Customized microscopy systems can now be built using freely available open-source software and open hardware designs, with significant reductions in both time and cost. Open hardware is a set of design principles and legal practices rather than a specific type of object and refers to hardware that uses free/open-source software. It can also refer to specific information provisions, such as outlines, designs, and implementations of hardware, as long as they carry a free license. Open-source software is defined as software in which source code can be used, modified, and redistributed by anyone regardless of whether it is for commercial or non-commercial purposes. In the field of light microscopy, several very well-known software systems are available under open-source licenses, for example, ImageJ [1], which is widely used for image analysis in scientific research and has become a standard analytic tool in biology. ImageJ has a user interface with easy-to-understand parameters for numerical calculations that may be used for various types of image processing tasks, and it is possible to perform calculation processing with high reproducibility based on pixel values.

Plugins written by users correspond to various image processing/analysis tasks. For example, Micro-Manager is also an open-source software and functions as a plugin for ImageJ [2]. It provides an easy-to-use interface for controlling microscope hardware and is used with many microscopes, CCD or CMOS cameras, and microscopy peripherals.

Confocal laser scanning microscopy (CLSM) has several advantages over conventional optical microscopy as it can obtain optical serial sections without mechanical specimen sectioning. CLSM markedly improves three-dimensional imaging characteristics [3,4,5]. The resolution obtained by conventional optical microscopes can be improved by about a factor of two within the exact focal plane. For a mathematical exposition of this method, see [4]. The advantage of confocal microscopy is the reduction of out-of-focus noises using pinhole or slit. Compared with the conventional optical microscope that uniformly irradiates light, the confocal laser scanning microscope uses the reflected light with less scattered light. It irradiates laser beam with high straightness at a short wavelength such as 488, 568, and/or 647 nm. By placing a pinhole at the position where the reflected light forms an image and limiting the laser path, it has the advantage of eliminating out-of-focus noises. Blurring caused by the light from the out-of-focus position of the focal plane of the objective lens is eliminated by the pinhole, so as to obtain a clear and high-contrast image. Another advantage of CLSM is the computer-aided three-dimensional reconstruction using serial optical sections. However, the confocal microscope equipment is considerably expensive.

Optical microtomography is possible without the mechanical processing of specimens. As optical noise due to scattered light is reduced, CLSM markedly enhances the contrast of the image. In addition to epifluorescence-type microscopes, CLSM can be applied to both inverted and upright optical path systems. Using thick whole-mount preparations and confocal microscopy alongside image reconstruction techniques, the three-dimensional organization of cytoskeletal components can be observed in cultured cell systems [6] and in endothelial cells in situ [7,8,9]; the glomerular structure of the olfactory bulb of a rabbit has also been studied using this method [10]. Conventional optical microscopes were designed to observe planar images spread on a focal plane by means of high-resolution optical lenses. The resolution depends on the numerical aperture of the objective lens, the wavelength of proof shielding, and the stable enclosure of the microscope body. According to Abbe’s theory, the minimum distance “d” at which two points of focus can be recognized as two images can be expressed by the following formula, where the wavelength of light is λ and the numerical aperture of the objective lens is NA

d = 1.22λ/2NA.

In his seminal 1873 paper, Abbe reported that the minimum resolution distance between two points using a conventional microscope was never less than half the wavelength of the imaging light [11]. For an oil immersion objective lens, if the NA is 1.2, the resolution is λ/2 on the focal plane—that is, the minimum distance between two resolvable points—will be about half the wavelength of the light being observed [12].

In addition to two-dimensional images, optical microtomography is also used to obtain three-dimensional information. It is possible to estimate structure in a direction perpendicular to the focal plane by moving the focal position up and down. As the focal position shifts, structures with different heights come into focus. However, the optical microscope has almost no capacity to convey information in the depth direction. Images in the depth direction captured by conventional optical microscopes are the result of recognition by the human brain. That is, although the observed image is composed of an almost infinite number of superimposed images of unfocused signals (noise) in the focused image, the human brain can act as an image processor to “picture” a clear image. In fluorescence microscopy, each fluorophore emits light over a range of wavelengths, causing nearby colors to overlap. A recent study demonstrated the presents a superpixel-based segmentation of muscle fibers in multi-channel microscopy in mice [13].

Another epifluorescence method called total internal reflection fluorescent microscopy (TIRFM) has attracted attention as an advanced observation method [14,15,16,17,18,19]. The main difference between CLSM and TIRFM lies in the extent of excitation: epifluorescence observation (including CLSM) involves irradiation of the entire excitation light in the direction of the optical axis and the subsequent observation of the fluorescence emitted, while TIRFM (which uses evanescent light, as described in the next paragraph) involves excitation within a very limited region of about 100 nm in the vicinity of the cover glass instead of the entire optical axis direction, and makes an observation of the resulting fluorescence of any internal reflection phenomenon. Using an excitation method such as TIRFM, it is possible to image reflection phenomena occurring only in the vicinity of the boundary surface and to detect fluorescent molecules with high sensitivity and with less background noise. The excitation light is made incident on a position deviated from the center of the objective lens’ microscope side by epi-illumination of the oil immersion objective lens and is reflected at the interface between the oil of the immersion lens and the cover glass. An evanescent field is thus formed at this boundary surface, and a thickness of 100 nm on the specimen side is illuminated. Therefore, fluorescence from the sample where it has been hit by the excitation light occurs only within this thickness range. Theoretically, assuming that the wavelength of incident light is 532 nm (e.g., the wavelength of a green diode laser) and the incident angle is 66°, the depth of penetration of the evanescent light into the immersion oil side generated at the interface between glass and water will be d = 106 nm. Using this technique, it is possible to excite substances in the vicinity of the cover glass in a very dark state with very little background optical noise. Again, due to this technique’s high specificity, it is possible to observe the behavior of a single fluorescent molecule, which is far beyond the resolution of optical microscopy. By the same token, however, the area that can be observed is limited to a distance of about 100 nm from the glass surface. Only one focal plane image can be detected. The major limitation of TIRFM is that, although biological samples are three-dimensional, TIRFM produces only a single two-dimensional image limited to the bottom of the sample. It is difficult to observe three-dimensional images using TIRFM [20].

Microscope autofocusing represents another recent advance that has been applied to conventional fluorescence microscopy. It can automatically and accurately adjust the focal position from the bottom to the top, making it a useful tool for photography and focal positioning. In particular, in long-exposure photomicrography, it is extremely difficult to maintain a constant focal position due to temperature changes and distortion occurring in the apparatus itself. It is necessary to adjust the focus from time to time, and the autofocus function becomes essential for such applications. However, microscopes with an integrated autofocus function are expensive and complex.

In the course of our research, we constructed a relatively simple and low-cost autofocus system using open-source software (Micro-Manager) and an open hardware system (pgFocus) developed by Karl Bellvé at the Biomedical Imaging Group (http://big.umassmed.edu/wiki/index.php/PgFocus) [21]. pgFocus monitors changes in focus through the positional changes of a reflected laser beam. It is designed to pass faster high-fidelity signals for Piezo *z*-axis controllers and add precise focus control signals to the controller.

The observation of optical cross-sections of fluorescently stained cells under a fluorescence microscope causes an overlap of out-of-focus images over against the in-focus plane when viewed from the eyepiece [22]. When observing a thick specimen by epifluorescence microscopy, a point spread function (PSF) shows how a bright spot that is sufficiently small (i.e., smaller than the wavelength of light) spreads in three dimensions to express the spread of light. The PFS is an important mathematical model representing the characteristics of the optical system. Using the PSF, the original object can be studied as a set of point light sources—that is, the light from all the point light sources spreads on the basis of the PSF—so that the images subsequently obtained are, in fact, mathematically calculated in a process known as deconvolution. In a sense, the microscope can be considered to work as a PSF generator to observe convoluted images alongside out-of-focus images.

The obtained images contain blurring due to the PSF, which is caused by noise in the unfocused portion or aberrations of the lens. However, the original image can be restored from such blurred images using an inverse calculation derived from the PSF. This process, known as “deconvolution,” corrects light behavior in the optical system, in such a way that the deconvoluted images can be modeled with sufficient accuracy [23]. The light distribution has specific characteristics for each lens, which can be expressed as the PSF for each lens system. The PSF can be obtained by measuring the light distribution unique to each lens by means of a visualizing fluorescent bead of fixed size. In this review, we describe a design for building a deconvolution microscope at low cost using readily available open-source software and open hardware. Although our system does not make use of expensive equipment, such as confocal microscopes or total internal reflection fluorescence microscopes, it allows good images to be obtained even without previous experience in electronics and optics. Deconvolution microscopy can yield good optical section images, thus making it possible to reconstruct three-dimensional images using open-source software systems. In addition, rotated images and tomographic images can be obtained. Deconvolution microscopy is a technique that eliminates the blur in the fluorescent microscope image. As optical tomograms are finally obtained, they are frequently compared with confocal laser microscopes. It is observed that the deconvolution processing of images taken with a confocal microscope is possible. Both confocal microscopy and deconvolution microscopy are fundamentally different technologies. The confocal microscope uses a pinhole to eliminate the light from outside the focus plane and obtain confocal images. Deconvolution microscopy restores the true three-dimensional luminance distribution of a whole specimen by calculation using computer-aided software. The advantages of this study are its cost-effectiveness and the ability to construct a microscope system using general-purpose commercially available optical components and open-source software.

In the present paper, we have described the performance of our deconvolution microscope system in preserving cytoskeletal components in cultured cells as a proof of concept.

When obtaining fluorescence microscopic images, extraneous fluorescence from regions other than the focal plane may overlap and result in an unclear image. Fluorescence outside the focal plane can be eliminated by either taking an optical section image using a CLSM or using a TIRFM to obtain a fluorescence image in a very narrow range from the adhesion surface. Although these methods may yield considerable improvements in image quality, the mixing of noise from elsewhere than the focal plane is frequently seen in CLSM, and TIRFM can only observe the portion adhering to the glass surface.

## 2. Standard Preparation of Fluorescent Staining with Cultured Cells for Deconvolution Microscopy

### 2.1. Antibodies and Fluorescent Reagents

Rhodamine-labeled phalloidin, Acti-stain 488 phalloidin (Cytoskeleton, Denver, CO, USA), and monoclonal anti-paxillin (BD Transduction Laboratories, San Jose, CA, USA) were purchased from the sources indicated. Polyclonal FITC-labeled goat anti-mouse IgG was purchased from BD Biosciences and used as the secondary antibody.

### 2.2. Cell Culture and Fluorescent Microsocpy

Fibroblasts (NIH3T3) was cultured in a 1:1 mixture of Dulbecco’s modified Eagle’s medium and nutrient mixture (F-12; Gibco, Grand Island, NY, USA), pH 7.4, containing 50 units/mL of penicillin, 50 mg/mL of streptomycin, and 10% fetal bovine serum (Gibco). The cells were maintained at 37 °C in a humidified 5% CO_2_ atmosphere. Cells were cultured on 35 mm glass-bottomed culture dishes (Matsunami Glass, Tokyo, Japan) overnight. For conventional fluorescence microscopy, samples were observed using an IX-70 inverted epifluorescence microscope with either a PlanApo X60 objective lens (1.4 NA, oil; Olympus, Tokyo, Japan) or an Apon X60 OTRF objective lens (1.17 NA, oil; Olympus, Tokyo, Japan).

### 2.3. Acquisition of Serial Sections Using Open-Source Hardware to Obtain Point Spread Function (PSF) Images

To move the objective lens using the Manager software system, we employed a method of automatically adjusting the focal position by driving a scanner for a Piezo objective lens and an objective lens with Piezo scanner (P-721; Physik Instrumente, Karlsruhe, Germany; from eBay around US $250–600) and Piezo Controller (E 610; Physik Instrumente, from eBay around US $50–100) [2,24]. The Piezo stage was controlled using a voltage controller (K8061; Velleman, Gavere, Belgium, from eBay around US $60).

A cooled CCD camera (CoolSNAP EZ; Photometrics, Tucson, AZ, USA) was used for conventional fluorescence imaging. We took photographs using the photo shooting function in the Micro-Manager software. The laser light for focal plane adjustment was supplied by a 488 nm argon ion laser attached to the total reflection microscope apparatus. Serial focal planes were obtained using the pgFocus software system.

The quality of an optical system can be defined as the extent of the spread of an object, also known as an object’s “blur.” For simplicity’s sake, we can take our object to be a single point. A three-dimensional blurred image of such a single point light source is usually called the PSF. In this study, we obtained serial optical sections of a microbead. Fluorescence images were taken continuously from the top, where the convoluted image first began to appear, to the bottom of the bead, where the convoluted light ended in the *z*-axis direction every 0.1 μm using fluorescent microbeads (Multiple Fluorophore Particles, SPHERO; Spherotech, Lake Forest, IL, USA) 0.1 μm in diameter. A series of captured images of a single bead (about 150 serial sections) were used as PSF images for deconvolution.

To obtain TIRFM images, a fluorescence microscope was combined with a total reflection microscope (TIRFM; IX2-RFAEVA; Olympus) to construct a conventional inverted fluorescent microscope (IX-70; Olympus). Photographs were taken and compared with both conventional and confocal microscopic images.

### 2.4. Deconvolution Serial Optical Sections Obtained by Epifluorescence Microscopy

In this study, we performed image processing by deconvolution using fluorescence of the cytoskeletal system in cultured cells using open-source software (μManager, NIH; ImageJ, NIH) and open hardware (pgFocus; University of Massachusetts, Boston, MA, USA). The system was applied to microscope observations. PSF images used for image processing were acquired using microbeads for each lens and fluorescent dye. Fibroblasts (NIH3T3) and skeletal myoblasts (L6) as specimens were stained with fluorescently labeled phalloidin (actin filaments) and anti-paxillin antibody (focal adhesions) as cytoskeleton markers after paraformaldehyde fixation. Continuous fluorescence microscope images in the *z*-axis direction were acquired by a combination of a scanner for the Piezo objective lens and pgFocus. The obtained PSF image and continuous fluorescence microscopic image were deconvoluted using ImageJ. Deconvoluted images were obtained using the Iterative Deconvolve 3D plugin for 2D and 3D deconvolution. This software is a plugin for 2D and 3D non-negative, iterative deconvolution, partially based on the DAMAS algorithm by Thomas F. Brooks and William M. Humphreys, Jr., NASA-Langley Research Center. It also includes a regularized Wiener filter as a preconditioning step, called DAMAS3 in aeroacoustics (https://www.optinav.info/Iterative-Deconvolve-3D.htm) [25].

## 3. Deconvolution Microscopy of Cytoskeletal Proteins Using Open-Source Software and Open Hardware

**Figure 1:**
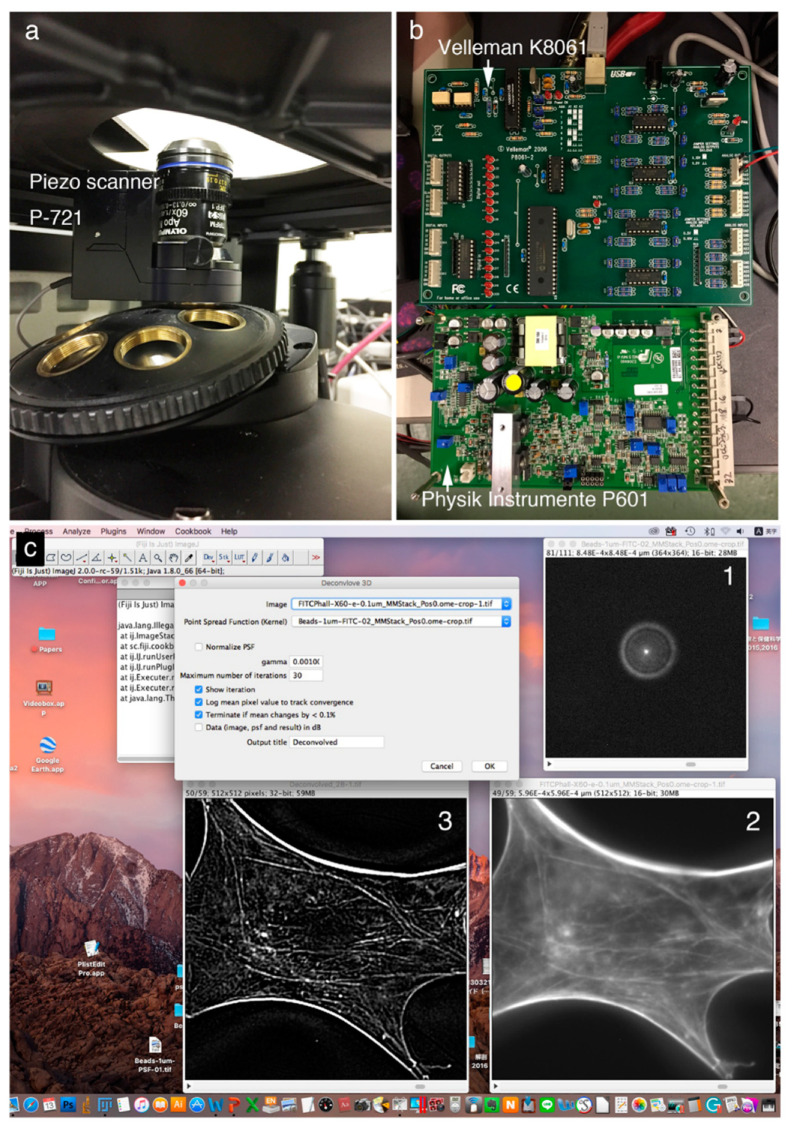
Deconvolution microscope system with a *z*-axis direction Piezo scanner stage with an objective lens (**A**), driver controller (**B**), and ImageJ software (**C**). Fluorescence microscope images were acquired using an objective lens scanner as a Piezo objective lens (A) and a voltage controller (B: Velleman K8061 and Physik Instrumente P601). The image under deconvolution processing is shown in Figure 1C. A PSF image prepared using microbeads (C-1), the fluorescence microscope image (C-2) before image processing, and the image after deconvolution image processing (C-3).

**Figure 2:**
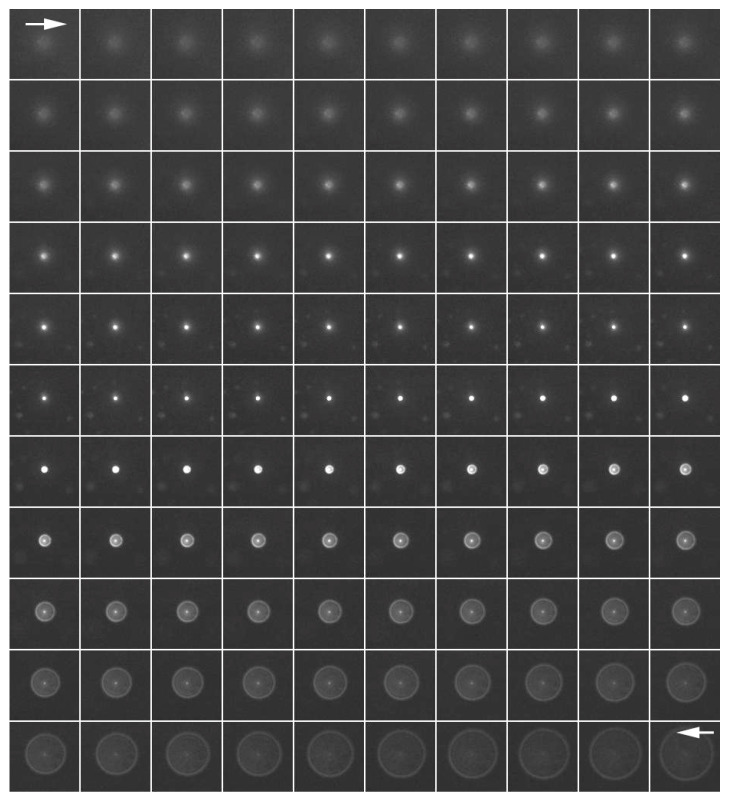
A point spread function (PSF) image acquired using fluorescent microbeads. Fluorescence images were taken continuously from the top to the bottom of the bead in the z-axis direction every 0.1 μm using fluorescent microbeads 0.1 μm in diameter. A series of captured images were used as the PSF image for deconvolution. The upper left arrow (→) shows the upper part of a bead image and the lower right arrow (←) shows the fluorescent images of the lower part of a bead image.

**Figure 3:**
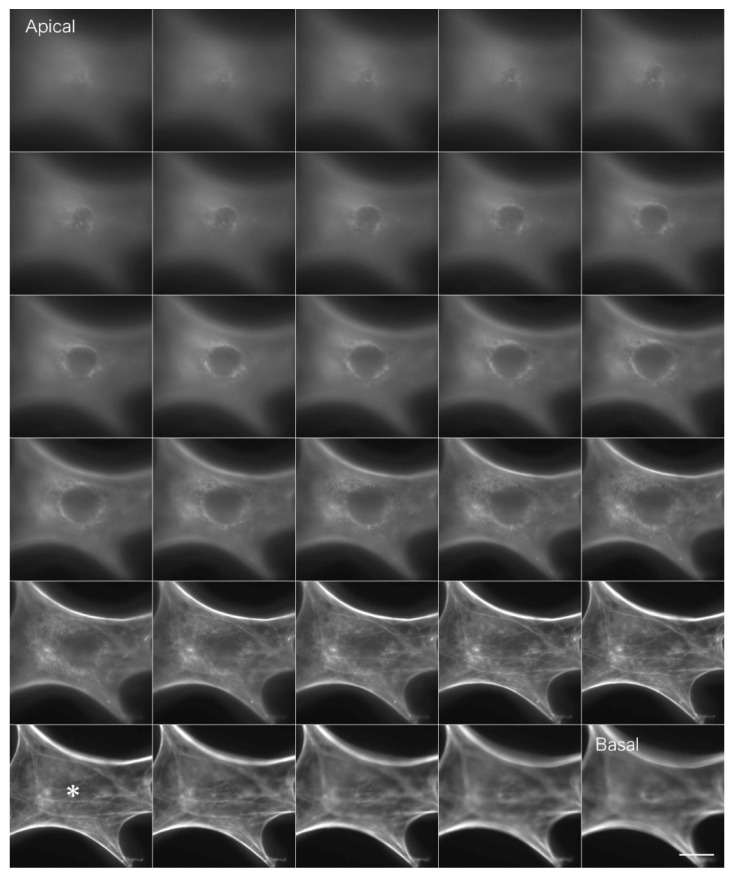
Conventional fluorescence microscopy of actin filaments in fibroblastic cells stained with FITC-labeled phalloidin under conventional fluorescence microscopy. Fibroblasts were fixed in formalin, and actin filaments were stained with FITC phalloidin. In conventional fluorescence microscopes, unnecessary fluorescence from outside the focal plane overlaps, resulting in the blurring of the convolution image. In particular, the fluorescent image on the basal plane is unclear due to overlapping out-of-focus fluorescence images from the upper surface layer (*; asterisk). Bar: 20 μm.

Figure 1 shows a deconvolution system with a Piezo scanner stage in the *z*-axis direction (Figure 1A), driver controller parts (Velleman K8061 and Physik Instrumente P601) (Figure 1B), and the ImageJ software system driving a Piezo objective lens (Figure 1C). Cases using a Piezo scanner (Figure 1A) for the objective lens and a voltage controller (Figure 1B: Velleman K8061) to drive the scanner and a driver controller for the scanner (Figure 1B: Physik Instrumente P601) are shown. Figure 1C shows the image under deconvolution processing by ImageJ. The figure shows the PSF image (Figure 1C-1) prepared using microbeads (φ 0.1 μm), the fluorescence microscope image before image processing (Figure 1C-2), and the image after deconvolution image processing (Figure 1C-3).

The PSF image was acquired using fluorescent microbeads (Figure 2). Fluorescence images were taken in the *z*-axis direction every 0.1 μm from the top to the bottom of a single fluorescent bead that was 0.1 μm in diameter. Deconvolution was performed using a series of captured images as the PSF image. The upper left arrow (→) shows the upper part of the bead and the lower right arrow (←) shows fluorescence images of the lower part of the bead.

Actin filaments in fibroblastic cells stained with FITC phalloidin were observed by conventional fluorescence microscopy (Figure 3). Briefly, fibroblasts were fixed in formalin, and actin filaments were stained with FITC phalloidin (Cytoskeleton). In a conventional fluorescence microscope, unnecessary fluorescence from outside the focal plane overlaps with the image, resulting in blurring. The fluorescence image on the basal plane is especially unclear due to out-of-focus fluorescence images from the upper surface layers (Figure 3*; see also Supplemental Information 1 for a serial optical image movie).

**Figure 4:**
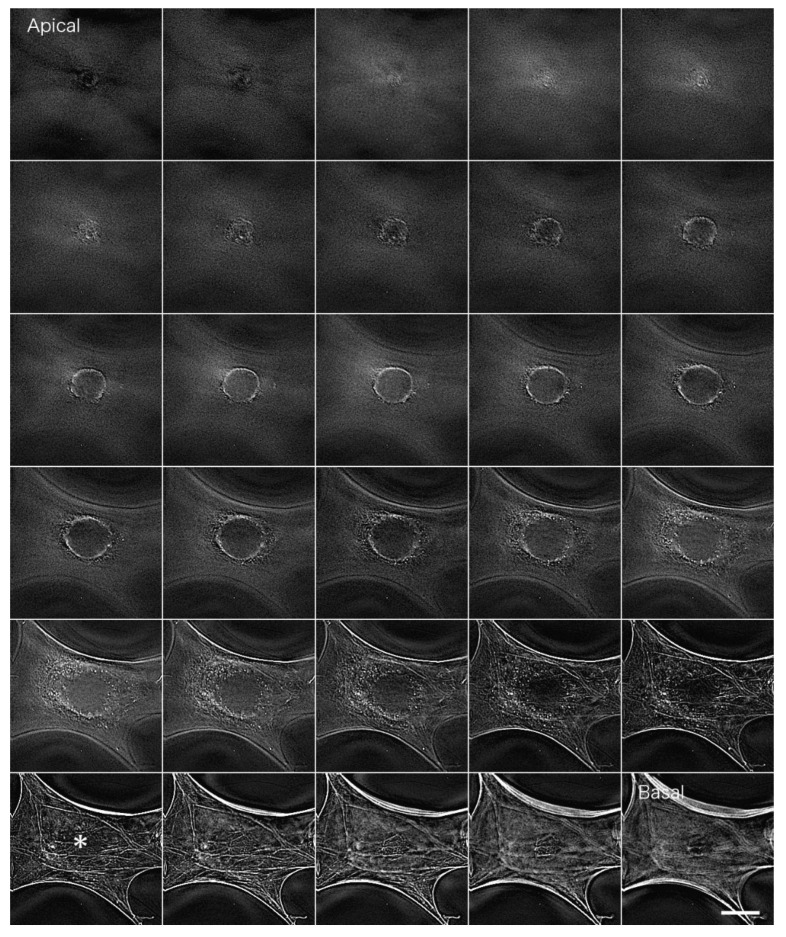
Actin filaments in fibroblastic cells stained with FITC phalloidin after deconvolution processing. The image acquired in Figure 3 was deconvoluted using a PSF image (Iterative Deconvolve 3D using ImageJ software). Out-of-focus fluorescence from regions other than the focal plane is removed. In particular, bundles of actin filaments localized on the basal plane of the cell (stress fibers) could be clearly detected (*; asterisk). Bar: 20 μm.

**Figure 5:**
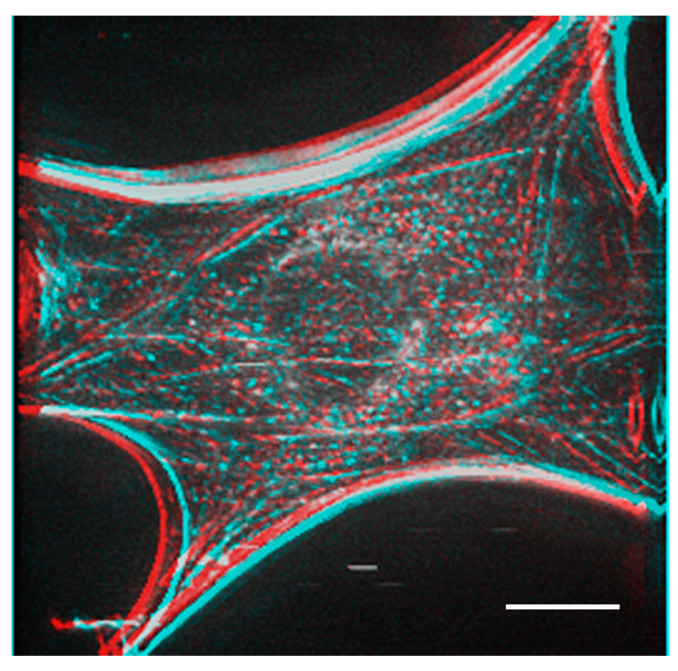
Three-dimensional reconstruction of actin filaments in fibroblasts.

Figure 4 shows actin filaments in fibroblastic cells stained with FITC phalloidin after deconvolution. We used a PSF image from the Iterative Deconvolve 3D program in ImageJ to deconvolute the image acquired in Figure 3. We removed out-of-focus fluorescence from regions other than the focal plane so that the bundles of actin filaments localized on the basal plane of the cell (stress fibers) could be detected clearly (Figure 4 asterisk; see also Supplemental Information 2 for a serial optical image movie). Figure 5 shows a stereo image generated using serial optical images of the deconvoluted image from Figure 4. The generated stereo pair image clearly showed the three-dimensional structure of the F-actin-based cytoskeleton. Anaglyph glass (red and blue) is required to view the stereo image. See also Supplemental Information 3 for a three-dimensional rotation movie.

A stereo image was generated from the actin fiber staining image after deconvolution obtained in Figure 4. Anaglyph glass is needed (red and blue) to see the stereo image. Bar: 20 μm.

**Figure 6:**
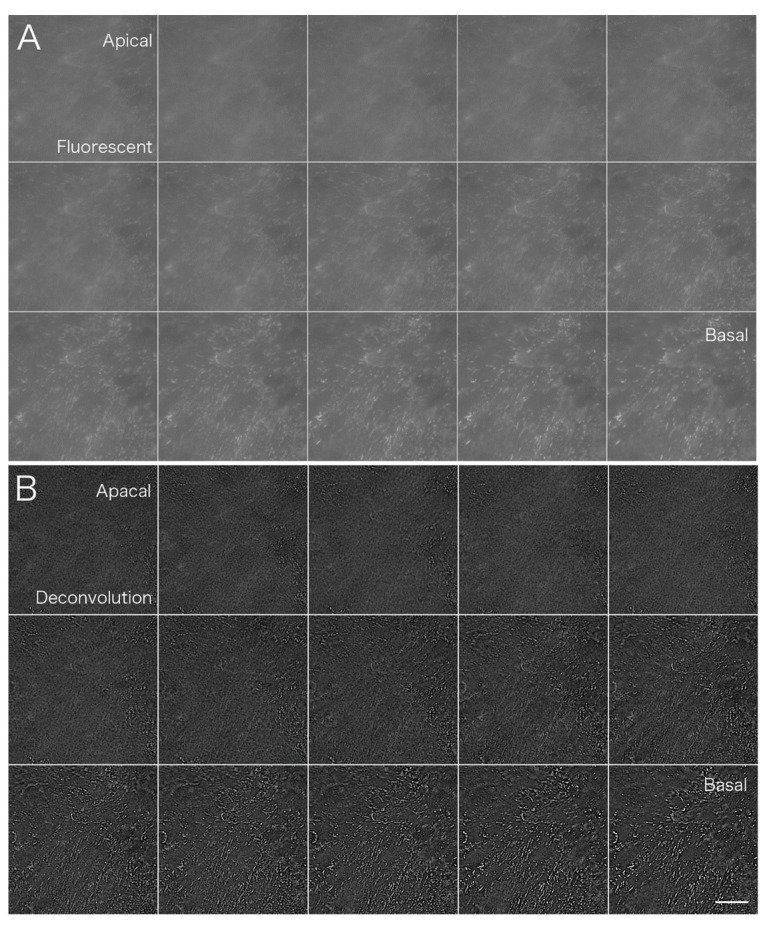
Paxillin in myoblast cells stained with anti-paxillin antibody. Myoblast cells were fixed with formalin, stained with anti-paxillin antibody, and observed by conventional fluorescence microscopy. Out-of-focus fluorescence from regions other than the focal plane overlapped, resulting in the blurring of the image. Particularly, out-of-focus fluorescence from the upper surface layer overlapped with the fluorescence on the basal plane (**A**: basal), which was unclear when cells were observed by conventional fluorescence microscopy (**A**). Deconvolution was performed using the image sequence obtained in (**A**) (Iterative Deconvolve 3D; ImageJ) and shown in (**B**). Out-of-focus fluorescence from regions other than the focal plane was removed almost completely; especially the focal adhesions localized on the basal plane of the cell could be clearly visualized (**B**: basal). Bar: 20 μm.

Myoblasts were fixed with formalin and stained with an antibody against paxillin, which is a marker for focal adhesions localized at sites of cell-substrate adhesion (Figure 6). Images were blurred by out-of-focus fluorescence from regions other than the focal plane. In particular, fluorescence from the upper surface layer overlapped with that from the basal plane, resulting in blurred images when cells were observed under conventional fluorescence microscopy (Figure 6A; see also Supplemental Information 4 for a serial optical image movie). Deconvolution was performed using Iterative Deconvolve 3D in ImageJ; the results are shown in Figure 6B (see also Supplemental Information 5 for a serial optical image movie). This procedure removed out-of-focus fluorescence from regions other than the focal plane almost completely, especially at focal adhesions localized on the basal plane of the cell.

**Figure 7:**
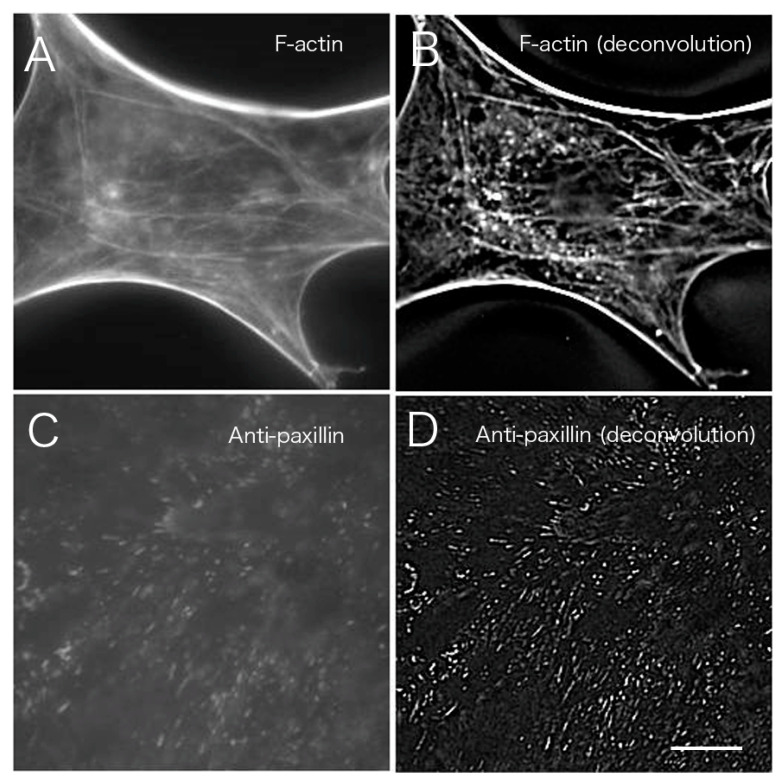
Comparison of deconvolution, CLSM, and TIRFM images. Images of phalloidin staining (**A** and **B**, enlarged images of Figure 3 asterisk and Figure 4 asterisk, respectively) were compared before (A) and after (B) image processing. Out-of-focus fluorescence other than the focal plane was markedly removed, resulting in a clear image. Anti-paxillin antibody staining (**C** and **D**) was also compared before (C) and after (D) deconvolution processing. Focal adhesions were detected more clearly after deconvolution compared to convolution images under conventional fluorescence microscopy. Bar: 20 μm.

Figure 7 shows a comparison of CLSM, TIRFM, and the images obtained after deconvolution. Images at the basal portion with phalloidin staining were compared before (Figure 7A) and after image processing (Figure 7B; Figure 3 asterisk, Figure 4 asterisk). Figure 7A,B are shown in higher magnification of Figure 3 asterisk (conventional) and Figure 4 asterisk (deconvolution). The results indicated a clear removal of out-of-focus fluorescence from outside the focal plane. The results of staining with an anti-paxillin antibody (Figure 7C,D) before deconvolution processing (Figure 7A) were compared with results from staining after deconvolution processing (Figure 7B). Focal adhesions were also clearly visible after the deconvolution procedure when compared to convolution images obtained by conventional fluorescence microscopy (Figure 7D).

## 4. Discussion

The confocal laser scanning microscope has a pinhole in the confocal plane in order to eliminate stray signals and to thereby enhance resolution in the optical axis (*z*-axis) direction in the context of high-contrast and high-resolution microscopy. In CLSM, light rays that excite the object converge to points that are in imaging relationship with the point light source from the laser light. Further, the point-like fluorescence generated in this focal plane is conveyed to the imaging point by the same optical system, so it is called a confocal scanning system. The system will have a resolution of λ/4, calculated as half of λ/2, which is the limit of resolution of the optical microscope. Moreover, the resolution in the optical axis direction could reach the limit of resolution along the focal plane of the conventional optical microscope. Confocal microscopy is applicable for observations of thick specimens and whole-mount preparations, but it requires equipment that is both complicated and expensive.

Under CLSM, the detection range of photomultiplier tubes (PMTs) used in the confocal microscope has limited the dynamic range due to the relatively low saturation power in a photocathode. Photocathode reflects the limitation of the signal-to-noise ratio as compared to the CCD or CMOS sensor used in this study. In CLSM, the detection range of photomultiplier tubes (PMTs) used in confocal microscopes is wider, but this increase in detection range is obtained by changing the gain width, which limits the direct intensity of light that can be detected. Within living cells, weak fluorescence in some focal planes may produce strong signals in some other areas. Such cases may not fit within the range of optical intensity detected by a PMT. For example, we captured the dynamics of actin protein using deconvolution microscopy with a higher resolution than CLSM images. For three-dimensional reconstruction, we were able to achieve resolution equivalent to or higher than CLSM using deconvolution images.

The blurring of concentric light in fluorescence microscopy cannot be avoided due to the fundamental characteristics of optical light from the specimen passing through the lens to form an image. Deconvolution technology returns the distribution of this optical light to its original position using mathematical operations and yields an image closer to the true original image. Deconvolution is used in fluorescence microscopy to gain improvements in the contrast of 2D and 3D images, 3D resolution, 3D rendering, visualization, etc.

Mathematical deconvolution processes are useful for the acquisition of high-contrast images, with improved image quality (i.e., finer details of processed images with high contrast), and they allow for better analysis due to the maintenance of quantification accuracy in deconvolution. Therefore, deconvolution is used as a preprocessing procedure for quantitative analysis, to track moving objects, and for measurements of area and brightness.

Deconvolution is also used to improve the signal-to-noise ratio, dynamic range, and contrast of resulting images, and to improve the axial resolution even with serial sections obtained by CLSM. Deconvolution techniques capture only the optical focal plane and exclude the light distributed outside the focal plane, and so are useful in determining the distribution of cytoskeletal proteins in cultured cells and thick tissues using computer-aided software systems. This technique allows clear images to be obtained even with weak fluorescent signals. CCD or CMOS cameras used in deconvolution can be used for detection over a wide range of signal strength, so images with a high number of detections of signal intensity range reflecting the strength of the signal obtained using a digital image sensor camera. On the other hand, confocal microscopy excludes diffused light outside the focal plane using hardware (i.e., a pinhole). However, deconvolution microscopy yields high-resolution images using software systems without the requirement for expensive hardware. PSF images can be obtained using small fluorophores and Piezo-derived or step motor-derived *z*-axis stages. It can be applied easily using open-source software, such as ImageJ (NIH), and the open hardware system pgFocus. 

To obtain optical sections using CLSM, it is necessary to set a large number of parameters, such as the scan speed and pinhole size. This variability means that the photographer must bring to bear a great deal of skill in order to set the appropriate conditions for any particular application. So, the image quality tends to differ depending on the researcher. On the other hand, the photography conditions when using deconvolution techniques are determined by the step size in the *z*-axis direction and the exposure time, so that it is easy to obtain images of consistent quality independent of the researcher’s skill. However, retaining image quality demands hardware precision, such as a microscope stage and lens that can be controlled with high precision in the directions of the x-, y-, and z-axes, and a high-quality lens with little aberration. For example, in time-lapse imaging, the temperature of the entire microscope must be kept constant. If the temperature of only a part of the system, such as the stage and lens, can be controlled, uneven temperature changes will lead to stage drift due to metal expansion and contraction. It is important to understand the influence of hardware precision on the images obtained and its importance in interpreting image data.

There have been major improvements in both the deconvolution technique and confocal microscopes, and appropriate microscopes can be selected for particular types of specimens. In our experience, confocal microscopy techniques are more suitable for thick tissues, while confocal microscopy seems to be more suitable for whole-mount preparations, such as blood vessels [8,9,26]. The microscope must be chosen according to the characteristics of the target. When staining multiple target proteins with fluorescent antibodies, and to observe live cells with reduced phototoxicity without damaging the cells at low excitation energies, a semiconductor light source and deconvolution technique should be used to accommodate a wide range of excitation wavelengths. Although this method is versatile, the thickness of the target is limited to about one cell layer (about 30 μm). Targets thicker than this will have too much light outside the focal plane, making it difficult to recover information from the original focal plane. In confocal microscopy, the light outside the focal plane is blocked by the pinhole, making this technique suitable for thicker samples, such as tissue slices or whole-mount preparations. However, confocal microscopy captures only light from the focal plane so that the researcher easily obtains serial optical sections from thick specimens. weaker signals may be lost during the acquisition of optical sections by CLSM. Increasing the power of the laser beam can increase the signal intensity, but this can result in cell damage.

The optimum observation system extracts the most information from each photon signal arriving from the specimen stained with fluorescent dyes. Thus, extracting the maximum information from each photon with single-photon signal excitation has aspects, one actually detecting each photon signal and that provides as much data from put focus noises. PMT detection with confocal microscopy is at least a common detector for comparably low signal detection. Both confocal and deconvolution microscopy using conventional fluorescence microscopes can detect real image photons revealing the 3D localization of cytoskeletal proteins. In addition, deconvolution microscopy can be used to simultaneously detect and record several types of information after deconvolution, such as fluorescent signals at different wavelengths, and can reduce back-scattered out-of-focus noise. Deconvolution microscopy using open-source software can be used to observe living cells and may be applicable for fluorescence microscopy in cell biology at a relatively low cost.

Although cell imaging technology is effective for research and analysis, and deconvolution provides useful results, accuracy may not be optimal due to the use of confocal microscope images. Microscope lenses always suffer from aberrations, and the images obtained vary according to lens performance and the temperature at the time of measurement. Therefore, in microscopy, it is necessary to understand that images will include these errors. There is a limit to the performance of the lens, and it is, therefore, necessary to take the limitations of the deconvolution technique and the confocal microscope into account when drawing conclusions from experimental results. Deconvolution processing is indispensable for the quantitative analysis of images. It is now possible to easily construct equipment for observation at a reduced cost using open-source software and open hardware designs. Deconvolution microscopy using open-source software and open hardware will become a powerful tool to detect cytoskeletal components using conventional fluorescence microscopes. In conclusion, the system described here demonstrates the ease of building a simple and efficient research-grade deconvolution microscope from inexpensive conventional stock devices, even with minimal experience in optics or electronics.

## Supplementary Materials

The following are available online at https://www.mdpi.com/2313-433X/5/12/88/s1. Supplemental_information_1.avi Serial optical images of conventional fluorescence microscopy of actin filaments. The movie shows serial optical images of actin filaments from the apex to the bottom of the cell. Supplemental_information_2.avi Serial optical images of actin filaments after deconvolution in a cell. The movie shows serial optical images of actin filaments after deconvolution from the apex to the bottom of the cell. Supplemental_information_3.avi 3D reconstruction of actin filaments in a cell. Deconvoluted images are projected to show the 3D distribution of actin filaments. Supplemental_information_4.avi Serial optical bestpart images of conventional fluorescence microscopy of paxillin. The movie shows serial optical images of paxillin from the apex to the bottom of the cell. Supplemental_information_5.avi Serial optical images of fluorescence microscopy after deconvolution of paxillin. The movie shows serial optical images after deconvolution of paxillin from the apex to the bottom of the cell.
